# Personalized Chronic Care for Diabetes Management in Pakistan: A Report of Two Cases

**DOI:** 10.7759/cureus.80577

**Published:** 2025-03-14

**Authors:** Safia Mehmood, Nashwa Kazmi, Sadia Khan

**Affiliations:** 1 Public Health, Meri Sehat Pvt. Ltd., Karachi, PAK; 2 Epidemiology of Cancer and Environmental Exposures (EPICENE) U1219, Bordeaux School of Public Health (ISPED) Bordeaux Population Health Center, Bordeaux, FRA

**Keywords:** adults, chronic disease, diabetes mellitus, dietary modification, glycemic control, lifestyle risk reduction, online intervention, preventive care, preventive health programs, wellness programs

## Abstract

This case report evaluates the efficacy of a personalized, multidisciplinary chronic care management (CCM) program aimed at improving health outcomes for individuals with polychronic conditions within Pakistan's under-resourced healthcare system. Implemented in November 2023, the intervention was designed to help two office employees newly diagnosed with type 2 diabetes and associated comorbidities, such as hypertension and obesity. Over six months, the participants engaged in a structured program that included teleconsultations, personalized care plans, and biweekly follow-ups through a social media application. The ‘care team,’ comprising doctors, nutritionists, wellness coaches, and care attendants, facilitated these interactions. Specific outcomes included a reduction in hemoglobin A1c (HbA1c) from 15% to 5.4% with a 26.3 kg weight loss for Participant 1 and a 3 kg weight loss with a decrease in HbA1c from 6.5% to 6.1% for Participant 2. This report highlights the potential of comprehensive, integrated, and patient-centered diabetes management to enhance glycemic control and weight management in settings burdened by high disease prevalence and limited healthcare resources.

## Introduction

The rising prevalence of chronic diseases such as diabetes, hypertension, and obesity has prompted healthcare systems globally to implement integrated models aimed at mitigating the cost and burden of chronic disease [1-3]. Currently, over 33 million adults in Pakistan are living with diabetes, a number projected to rise sharply due to factors like genetic predisposition, increasing urbanization, and lifestyle changes such as dietary shifts toward more processed foods, reduced physical activity, etc. The International Diabetes Federation highlights Pakistan as one of the countries with the highest burden of the disease globally, where the prevalence and impact of diabetes continue to increase alarmingly [4].

Resource constraints, inconsistent governance, and inadequate healthcare policies further complicate the effective management of diabetes in Pakistan [5,6]. This case report describes a personalized chronic care model developed for two patients newly diagnosed with type 2 diabetes, highlighting how telemedicine, health coaching, and ongoing monitoring were utilized to improve glycemic control, reduce weight, and enhance quality of life. The intervention showcases the potential of integrated, patient-centered approaches in improving diabetes care in settings with limited healthcare resources.

Evidence from similar low-resource settings suggests that chronic care models (CCMs) can improve health outcomes. Studies have demonstrated significant improvements in glycemic control and quality of life factors that include weight management and reducing complications associated with multiple comorbidities due to the implementation of strategies such as self-monitoring of behaviors and mobile health applications, web-based interventions, patient-centered care for self-management, lifestyle modifications, online one-on-one health coaching, and telemedicine [7-11]. For instance, a 2023 study by BeatO (Health Arx Technologies Pvt. Ltd., Delhi, India) demonstrated that a digital diabetes care program incorporating personalized coaching and digital monitoring tools effectively improved glycemic control and weight loss, underlining the value of accessible, continuous care strategies [12].

## Case presentation

In August 2023, two male office employees of Meri Sehat Pvt. Ltd., aged 33 and 26, were diagnosed with type 2 diabetes mellitus. Participant 1, aged 33, presented with uncontrolled type 2 diabetes mellitus, evidenced by a high hemoglobin A1c (HbA1c) level of 15% and additional complications of hypertension, asthma, and obesity. His treatment regimen included multiple medications, notably two different types of insulin: Humulin R, administered three times daily, and Toujeo, taken at bedtime, to manage his blood glucose levels effectively. He had also been prescribed antihypertensives, lipid-lowering agents, and asthma control medications, reflecting the complexity of managing his polychronic conditions. Participant 2, aged 26, was also diagnosed with type 2 diabetes with an HbA1c level of 6.5%. Both participants experienced weight gain and significant distress due to early-onset chronic conditions and faced challenges with self-management, support systems, and access to expert guidance. Detailed demographic characteristics are highlighted in Table 1.

**Table 1:** Participant demographics HbA1c: hemoglobin A1C; HS: at bedtime; OD: once daily; BID: twice a day; TDS: three times a day; SOS: if necessary, as needed; MG: milligrams; tab: tablet; Inj.: injection

Demographic and Clinical Information	Participant One	Participant Two
Age	33 years	26 years
Gender	Male	Male
Concerns	Type 2 diabetes (HbA1c 15.0% on August 14, 2023), weight gain, sedentary lifestyle	Type 2 diabetes (HbA1c 6.5% on Dec 4, 2023), weight gain, sedentary lifestyle
Comorbidities	Hypertension (high blood pressure), asthma, obesity	Overweight
Medications	Tab. Advant 8 MG x HS (at bedtime), Tab. Tenormin 25MG x OD (once daily), Tab. Rovista 10mg x HS (at bedtime), Inj. Humulin R x s/s x s/c x TDS, Inj. Toujeo x 20 units x HS (at bedtime), Tab. Neurobion x OD (once daily), Montelukast x HS, foster inhaler x BD, ventolin inhaler x SOS	None
Goals	Weight loss, increased physical activity, decreased HbA1c levels	Weight loss, increased physical activity, decreased HbA1c levels

This led to the development of a chronic care management program designed by our organization's Founder/Chief Executive Officer (CEO), Director of Public Health, and Manager of Public Health. The program was implemented from November 2023 to May 2024, with data collection running from December 6, 2023, to May 31, 2024, allowing for both initial assessments and follow-ups. The intervention was carried out by a ‘care team’ consisting of Pakistan Medical and Dental Council (PMDC)-certified care managers, care attendants, and a wellness coach. The project was funded by Meri Sehat Pvt. Ltd. and supported by Essa Labs (Pakistan), which provided discounted at-home testing services.

Our intervention, informed by a review of 198 studies on hypertension and diabetes in low-and middle-income countries (LMICs), emphasized multifaceted approaches with self-management support, patient education, and coordinated healthcare teams [13]. The CCM has demonstrated significant benefits in managing type 2 diabetes, especially when incorporating key components like self-management support and decision support. The 2022 American Diabetes Association (ADA) Standards of Medical Care in Diabetes endorse a CCM-based approach, emphasizing strategies such as collaborative goal setting, integration of evidence-based guidelines, and addressing barriers to care [14]. Additionally, research on South Asian populations in the UK highlights the effectiveness of culturally tailored dietary interventions for weight loss and diabetes remission [15]. This approach enabled us to offer personalized, culturally sensitive care and fill gaps in continuous care. A cost-benefit analysis demonstrated that replicating a similar multidisciplinary team (MDT) support system independently would be costly (>PKR 100,000) for the participants [16]. Hence, as a pilot intervention, the program was offered free of cost (FOC) to both participants.

The personalized chronic care management program was structured around several key components. It began with comprehensive initial assessments, including blood tests, body measurements (tape measures of chest, upper abs, lower abs, glutes, biceps, thighs, calves, and shoulders, all in inches), and a three-day food diary. These informed personalized ‘care plans’ were tailored to each participant's specific health needs. These care plans included dietary guidelines, physical activity recommendations, and emergency management strategies for diabetes and comorbid conditions. Health metrics such as blood glucose levels and weight were monitored on a daily and weekly basis, enabling ongoing adjustments to the care plans. Participants were also referred to specialists and discounted medication services as required. Care continuity was ensured through fortnightly follow-ups conducted by the multidisciplinary care team. Additionally, the program included weekly panel discussions among the team to review and adjust the care plans based on the latest health status updates and participant feedback. Regular monitoring of health outcomes was supported by pictorial proof, ensuring accurate tracking and documentation of each participant's progress.

The inclusion criteria were targeted towards adults diagnosed with chronic conditions such as diabetes, hypertension, or obesity, particularly those experiencing significant management challenges or diabetes distress. Conversely, the exclusion criteria were set to omit individuals with acute or terminal conditions, severe mental health disorders that impede participation, active substance abuse, or those lacking consent. This approach ensured the program was tailored to participants who benefited most from a structured and supportive care environment while maintaining ethical and practical boundaries.

The evaluation used quantitative methods to assess program effectiveness through descriptive statistics and time series charts. Data collection utilized Google Forms (Google LLC, Mountain View, California, United States) with Excel analysis. Participant 1 experienced a weight loss of 26.3 kg and a decrease in HbA1c by 9.6% (Table 2), which allowed them to transition from insulin to oral hypoglycemics. Participant 2 achieved a weight loss of 3 kg and a reduction in HbA1c from 6.5% to 6.1%, as noted in Table 2.

**Table 2:** Summary of key health outcomes HbA1c: hemoglobin A1C; a: November 30, 2023; b: May 31, 2024

Participant	HbA1c (%)-(Start of Intervention^a^)	HbA1c (%)-(End of Intervention^b^)	Weight (kg)-(Start of Intervention)	Weight (kg)-(End of Intervention)
One	15.0	5.4	113.5	87.2
Two	6.5	6.1	75	72

**Figure 1:**
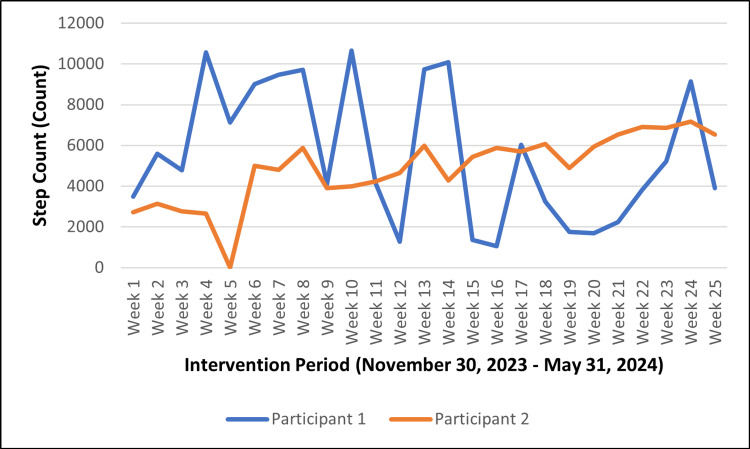
Weekly step count trends for participants over the intervention period (November 30, 2023 – May 31, 2024)

Diet plan compliance was assessed based on the following criteria: Yes, fully (90-100%), mostly (70-89%), somewhat (50-69%), and not at all (less than 50%). Both participants were “mostly” compliant with the care plan, i.e., 70-89% of the time, as self-reported by the participants. Both participants were assigned a daily target of 10,000 steps. On average, they achieved approximately 5,221 steps per day. Figure 1 depicts the step count trends observed during the intervention period. 

Additionally, both participants reported qualitative improvements such as enhanced quality of life, citing improved decision-making abilities, reduced diabetes-related distress, increased energy levels, and better sleep quality. Other health issues, such as blood blisters, acne, toenail removal, and neuropathy, were also successfully addressed through timely medical referrals.

The study was conducted under Meri Sehat Pvt. Ltd.'s Institutional Review Board (IRB00014486) approval, with written informed consent obtained from all participants.

## Discussion

Our results align with global findings from chronic care model (CCM) implementations, which consistently emphasize person-centered care and patient empowerment [14]. Our findings and observations reflected the positive outcomes observed globally, such as increased patient engagement, improved self-management, and better clinical results [17,18]. Additionally, studies have shown that remote monitoring and real-time communication with healthcare providers significantly improve HbA1c levels, strengthen diabetes self-care, and support sustainable behavior change in patients with type 2 diabetes mellitus (T2DM), mirroring the improvements observed in our intervention [19].

Key strengths of our intervention include its human-centered design, which focused on building rapport between the care team and the participants, and the telemedicine model that provided convenient access to a dedicated care team. While the outcomes were positive for Participant 1, Participant 2 showed limited weight loss (3 kg) and a modest HbA1c reduction of 0.4% due to inconsistent adherence to the program, reflecting real-world challenges with regard to compliance. Further research with larger, more diverse populations could help validate these findings and explore the potential for wider implementation in comparable settings.

## Conclusions

This case report demonstrates that personalized care management, with its focus on holistic patient care, can lead to significant improvements in diabetes outcomes. The improvement in Participant 1's HbA1c levels and weight, along with the moderate changes observed in Participant 2, suggests the potential value of comprehensive, individualized care approaches in managing chronic conditions. The success of the intervention has led to word-of-mouth dissemination, resulting in a substantial increase in participant enrollment. The findings from this expanded cohort will be analyzed and published in due course.
